# Comparative Effects of Bone Marrow Mesenchymal Stem Cells on Lipopolysaccharide-Induced Microglial Activation

**DOI:** 10.1155/2013/234179

**Published:** 2013-03-24

**Authors:** Fan-Wei Tseng, May-Jywan Tsai, Li-Yu Yu, Yu-Show Fu, Wen-Cheng Huang, Henrich Cheng

**Affiliations:** ^1^Department and Institute of Pharmacology, School of Medicine, National Yang-Ming University, Taipei 11221, Taiwan; ^2^Neural Regeneration Laboratory, Department of Neurosurgery, Neurological Institute, Taipei Veterans General Hospital, Taipei 11221, Taiwan; ^3^Center for Neural Regeneration, Neurological Institute, Taipei Veterans General Hospital, Taipei 11221, Taiwan; ^4^Department of Anatomy and Cell Biology, School of Medicine, National Yang-Ming University, Taipei 11221, Taiwan; ^5^Department of Education and Research, Taipei City Hospital, Taipei 11221, Taiwan; ^6^Faculty of Medicine, School of Medicine, National Yang-Ming University, Taipei 11221, Taiwan; ^7^Institute of Brain Science, School of Medicine, National Yang-Ming University, Taipei 11221, Taiwan

## Abstract

After injury to the CNS, microglia are rapidly activated and concentrated and trigger inflammatory reaction at the sites of injury. Bone marrow mesenchymal stem cells (BMMSC) represent attractive cell sources for treating CNS injury. Although anti-inflammatory and paracrine effects of grafted BMMSC have been shown, direct modulation of BMMSC on microglia *in situ* remains unclear. The present work employs *in vitro* transwell assay to characterize the effects of BMMSC on LPS-stimulated microglia. BMMSC are cultivated in serum and serum-free (sf) conditions, namely, BMMSC and BMMSC-sf. Both cultures express major surface markers specific for mesenchymal stem cells. However, the BMMSC-sf exhibit sphere-like structure with reduced expression of two adherent cell markers, CD29 and CD90. Compared to BMMSC-sf, BMMSC are fibroblast like and have faster differentiation potential into neural-like cells. Furthermore, BMMSC release significant levels of TIMP-1 and VEGF, regardless of being alone or in coculture. The downregulated MMP-9 mRNA may be caused by TIMP-1 secretion from BMMSC. Our cell culture system provides a powerful tool for investigating the molecular and cellular changes in microglia-BMMSC cocultures.

## 1. Introduction

Microglia, CNS-resident macrophages, play important roles in the physiological and pathological conditions of the central nervous system (CNS). After injury to the CNS, microglia are rapidly activated and concentrated and trigger inflammatory reaction at the sites of injury [1, 2]. Ample evidence has shown that activated microglia contribute to destructive processes leading to secondary neuronal degeneration. The responses in activated microglia include morphological changes, migration [3], proliferation [4], nitric oxide (NO) production, phagocytosis, antigen presentation, and secretion of diffusion factors. Activated microglia also released excess of toxic factors (such as TNF-*α*, IL-1*β*, superoxide, and NO) [5, 6].

Neurorestorative therapy with BMMSC is a promising treatment for CNS injury. BMMSC are found to exhibit low immunogenicity and can escape recognition by lymphocytes and natural killer cells. This distinguishing feature makes the match of BMMSC between donors and recipients less restricted than other cells. In addition, BMMSC can be isolated from bone marrow with relative ease. These multipotent cells also have the ability to differentiate into other types of cells. Several beneficial effects of BMMSC have been proved, including neuron protection [7], differentiation in the lesion site [8], and increased proliferation of endogenous neuron stem cells [9]. These findings support the potential utility of BMMSC for cell-based therapeutic applications. Given that BMMSC behave as biomolecular factories, the approaches for CNS injury treatment shall be further developed. However, there has been much controversy regarding therapeutic mechanisms and potential risks of various BMMSC activities in the injury sites. The culture media supplements are important issues to discuss. Fetal bovine serum (FBS) is generally supplemented in a complete media for* ex vivo* expansion of BMMSC. However, FBS might have contamination risks with unknown factors or prion which cause Creutzfeldt-Jakob disease (CJD) in humans. In order to accomplish successful cell therapies for CNS injury patients, it is critical to consider and avoid animal serum contaminations.

Several lines of evidence have shown that BMMSC therapeutic plasticity relies greatly on the paracrine release of molecules. However, the direct modulation of BMMSC to the endogenous immune cells of CNS, microglia, is not yet clear. In the present study, BMMSC, cultivated in serum and serum-free (sf) conditions, and an *in vitro* model of microglia-BMMSC cocultures are employed. The effectiveness of BMMSC on LPS-induced microglial activation and cytokine expression is examined and compared. Our results suggest that BMMSC release factors and exert modulation on microglia in a cell contact-independent communication.

## 2. Material and Methods

### 2.1. Materials

Culture multiwells and pipettes were obtained from Orange Scientific (Graignette, Belgium). Cultured media, fetal bovine serum (FBS), and antibiotics were purchased from Gibco (Invitrogen Corporation, USA). A rat cytokine array was purchased from R&D (ARY008). Cell surface antibodies for cytometric analysis were from BD Bioscience (USA). Lipopolysaccharide (LPS; *Escherichia coli *O111:B4) was purchased from Sigma-Aldrich (St. Louis, MO,USA). Other reagents were purchased from Sigma-Aldrich unless stated otherwise.

### 2.2. Microglia Culture

Microglial cells were isolated from confluent mixed glial cell cultures as described previously [10, 11]. Briefly, floating cells and weakly attached cells on the confluent mixed glial cell layer [12, 13] were isolated by shaking the flasks for 2 hrs at 180 rpm. The resulted cell suspension was transferred to culture dish and allowed to adhere at 37°C. Unattached cells were removed after 30 min. Microglia were isolated as strongly adhering cells. The enriched microglial cultures were 99% positive for OX-42 (Serotec' MCA275R) as assessed by immunostaining. Microglia were nonstimulated or stimulated by LPS (100 ng/mL) for 24 hours before coculture with BMMSC. After microglial activation, the cells were fixed for immunostaining and the medium was collected for NO release assay.

### 2.3. BMMSC and BMMSC-sf Culture

Bone marrow cells were isolated and prepared from femurs of young adult male Sprague-Dawley (SD) rats. Briefly, four-week-old SD rats were sacrificed by injection of sodium pentobarbital. Femurs were dissected from the attached musculature and connective tissues. Bone marrow cells were flushed out from femurs with phosphate buffered saline (PBS; GIBCO) and filtered through 70 *μ*m pore sieve. The filtered cells were collected by centrifugation (326 ×g for 10 minutes), resuspended, and maintained as monolayer cultures in Dulbecco's modified Eagle's medium/F12 (DMEM/F12; Invitrogen, Carlsbad, CA, USA), supplemented with 1% penicillin/streptomycin and 10% fetal bovine serum (FBS) at 37°C in a humidified atmosphere with 5% CO_2_/95% air. Cells growing in such condition throughout 0–5 passages were designated as BMMSC. When BMMSC grew to 80% confluence at passage 0, cells were washed twice and switched to serum-free media containing DMEM/F12 supplemented with 2% B27 (Invitrogen), bFGF (20 ng/mL), and EGF (20 ng/mL). Numerous cells would suspend in serum-free medium and aggregate to form neurosphere-like mass within 7–10 days. The cellular mass was collected and replated to new culture flasks under the same condition for cell expansion, designated as BMMSC-sf. Both BMMSC and BMMSC-sf were expanded and subcultured at least for 5 passages. Phenotypic characterizations of cultures were examined by immunostaining and by flow cytometric analysis against cell surface markers. Furthermore, both cultures were processed for multilineage differentiation assays, including adipogenesis and neuronal differentiation.

### 2.4. Coculture Assay

Microglia were harvested from flasks and seeded 1 × 10^6^ cells/well in 6-well plate in low glucose DMEM/F12 medium containing 10% FBS and 1% penicillin/streptomycin in the presence of absence of 100 ng/mL LPS. Approximate 3 × 10^5^ cells/well of BMMSC or BMMSC-sf were seeded to the transwell inserts (1 *μ*m Millicell PET membrane, Millipore) for 24 hours. The transwell inserts were then replanted on top of microglia and further cocultured for 6 or 24 hours. Conditioned media of cultures or cocultures were then collected for the analysis of ELISA, protein array, and western blot. Furthermore, the microglia after coculture with BMMSC or treatment were harvested for total RNA extraction and processed for Q-PCR analysis.

### 2.5. Flow Cytometric Analysis of Expressed Antigens on Cell Surface

The specific surface markers of isolated and expanded cells were detected at passages 0 to 5 by flow cytometric analysis. Briefly, BMMSC and BMMSC-sf were harvested by treatment of 5 mM EDTA in PBS solution. The cells were stained for 1 hour on ice with fluorescein isothiocyanate- (FITC-) or phycoerythrin- (PE-) conjugated antibodies for cell surface markers, including CD34 (hematopoietic lineage early marker), CD90 (Thy-1), CD44, CD54, and CD29 (integrins) (BD bioscience). The stained cells are analyzed by fluorescence-activated cell sorter (FACS Calibur flow cytometer; BD bioscience) using a 525 nm bandpass filter for green FITC fluorescence and a 575 nm bandpass filter for red PE fluorescence.

### 2.6. Cell Differentiation

A commercial kit (Mesenchymal Stem cell Adipogenesis kit, Chemicon SCR020) was employed for *adipocyte differentiation* of our cultured BMMSC and BMMSC-sf. Following the instructions of the kit, cultures were grown in adipogenic induction medium for 72 hrs and then replaced with adipogenic maintenance medium for 24 hrs. The replacement of the media was repeated for three times. Finally, the cells were cultured for one additional week with adipogenic maintenance medium. The adipocytes were identified by staining with oil red solution for oil drops and counterstaining with hematoxylin solution. For *neural differentiation, *BMMSC and BMMSC-sf were harvested from cultured flasks. Equal cell numbers of BMMSC and BMMSC-sf were seeded onto poly-L-lysine-coated 24-well plate in DMEM/F-12-based differentiation medium containing 2 mM L-glutamine, 20 ng/mL bFGF, 30 ng/mL BDNF, and 5% FBS. The medium was refilled every 3 days. Cells were fixed at days 3, 7, and 14 of differentiation for immunocytochemistry.

### 2.7. Immunocytochemistry Analysis

Cultured cells were fixed with 4% paraformaldehyde for 30 min. Cells were further permeabilized with 0.1% Triton X-100, blocked with 1% bovine serum albumin, and immunostained with primary antibodies and with the respective fluorescently tagged secondary antibodies (Jackson ImmunoResearch Inc.). Primary antibodies included mouse anti-GalC (Chemicon, USA), mouse anti-ED-1 (1 : 200), mouse anti-inducible nitric oxide synthase (iNOS) (BD Bioscience, USA), and rabbit anti-*β*III tubulin (Covance, USA). Images of cultured cells were obtained with a fluorescent microscope equipped with fluorescence optics and with a CCD camera. Micrography was performed using a 10X and 20X objective, and images were processed with imaging software (MetaMorph Imaging System, Universal Imaging Corp, Downingtown, PA, USA).

### 2.8. Biochemical Assays

The production of nitric oxide (NO) was assayed as the accumulation of nitrite in the medium using colorimetric reaction with Griess reagents (1% sulfanilamide/0.1% naphthylethylenediamine dihydrochloride/2% phosphoric acid) as described by Tsai et al. [14]. After LPS treatment, the culture medium was collected, mixed with Griess reagents, and incubated at room temperature for 10 min. The absorbance of the resultant products was measured at 540 nm. Sodium nitrite (NaNO_2_) was used as the standard to calculate nitrogen dioxide (NO_2_) concentrations. A rat protein cytokine kit (R&D, ARY008) was used to screen the expression of 29 rat cytokines in the released fractions (media) of coculture. The levels of cytokine expression were determined by the intensity of immunoreactivity, relative to that of the standard controls, following the manufacturer's instructions. TIMP-1 level in the coculture medium was further identified by using a TIMP-1 ELISA Kit (RayBio ELR-TIMP1-001). The level of TIMP-1 expression was determined by the intensity of optical density 450 nm, following the manufacturer's instructions.

### 2.9. Real-Time PCR

Total RNA was extracted using TRIzol kit (Invitrogen). RNA was reverse transcribed in a final volume of 20 ul using 0.5 ug of oligo-dT and 200 U Superscript III RT (Invitrogen) for 30 minutes at 50°C, followed by 2 minutes at 94°C to inactivate the reverse transcriptase. Polymerase chain reaction (PCR) amplification of the resulting cDNAs was performed under the following conditions: 35 cycles of 94°C for 30 seconds, 58°C for 45 seconds, and 68°C for 45 seconds, in which the 68°C step was increased by 5 seconds every cycle after 10 cycles. For real-time PCR, the amplification was carried out in a total volume of 10 ul containing 0.5 uM of each primer, 4 mM MgCl_2_, 1 ul of LightCycler FastStart DNA Master SYBR green I (Roche Molecular Systems), and 5 ul of 1 : 20-diluted cDNA. The primers and sequences were iNOS (forward: AAG, AGA, CGC, ACA, GGC, AGA, G; reverse: CAG, GCA, CAC, GCA, ATG, ATG, G), IL-1*β* (forward: TCA, AAT, CTC, ACA, GCA, GCA, TCT, CG; reverse: ACA, CTA, GCA, GGT, CGT, CAT, CAT, CC), TNF-*α* (forward: GCC, GAT, TTG, CCA, CTT, CAT, AC; reverse: GGA, CTC, CGT, GAT, GTC, TAA, GTA, C), Arg-1 (forward: TTG, ATG, TTG, ATG, GAC, TGG, AC; reverse: TCT, CTG, GCT, TAT, GAT, TAC, CTC, C), and IL-4 (forward: CGT, CAC, TGA, CTG, TAG, AGA, GC; reverse: GGG, CTG, TCG, TTA, CAT, CCG), IL-10 (forward: CAC, TGC, TAT, GTT, GCC, TGC, TCT, TAC; reverse: GGG, TCT, GGC, TGA, CTG, GGA, AG), MMP-9 (forward: TGT, ATG, GTC, GTG, GCT, CTA, AAC; reverse: AAG, GAT, TGT, CTA, CTG, GAG, TCG), and RPL-13 (forward: AGG, TGG, TGG, TTG, TAC, GCT, GTG; reverse: GGT, TGG, TGT, TCA, TCC, GCT, TTC, G). PCR reactions were prepared in duplicate and heated to 95°C for 10 minutes followed by 40 cycles of denaturation at 95°C for 15 seconds, annealing at 60°C for 1 minute and extending at 72°C for 20 seconds. Standard curves (cycle threshold values versus template concentration) were prepared for each target gene and for the endogenous reference (ribosomal protein L13A (RPL13)) in each sample. The quantification of the unknown samples was performed using the _ΔΔ_Ct converting formula.

### 2.10. Western Blot Analysis

The following antibodies were used for western blot analysis: goat anti-TIMP-1 (1 : 1000; Santa Cruz SC-6832) and rabbit anti-VEGF (1 : 1000; Abcam). The membranes were blocked with 5% nonfat milk in PBS-T for 1 h at room temperature and then incubated with primary antibodies overnight at 4°C. The membranes were then processed with HRP-conjugated secondary antibodies. Immunoreactive bands were visualized using chemiluminescence ECL western blotting detection reagents (Amersham, Piscataway, NJ, USA). Experiments are performed in duplicate to ensure reproducibility. Ponceau-S staining was used for internal control.

### 2.11. Statistical Analysis

Experimental data were expressed as the mean of independent values ± SEM and were analyzed using one-way analysis of variance (ANOVA) followed by Bonferroni's *t*-test. Values of *P* < 0.05 were considered to show statistical significance.

## 3. Results

**Figure 1:**
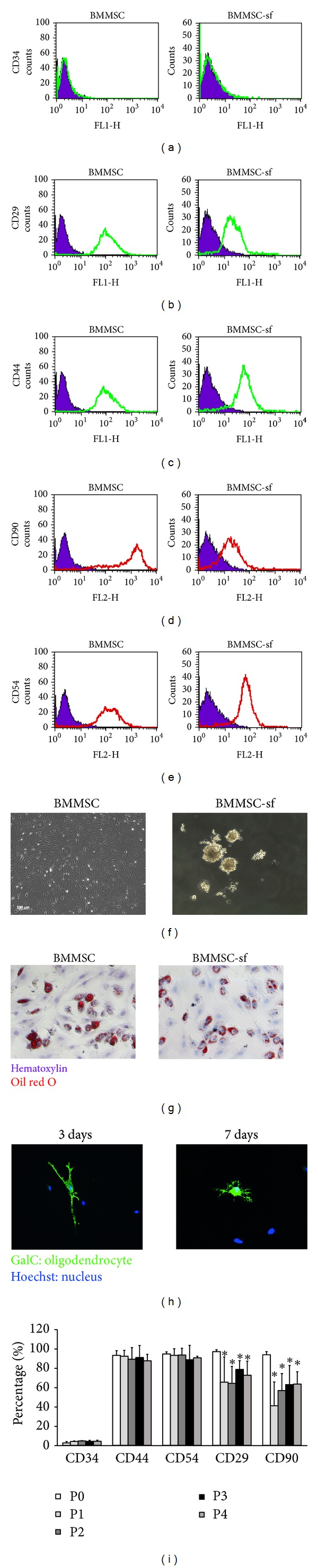
Characterization and flow cytometry of bone marrow mesenchymal stem cells (BMMSC) during expansion in serum-free (sf) or serum-containing medium. ((a)–(e)) Comparative flow cytometry analysis of cell surface markers in BMMSC or BMMSC-sf. (f) Phase-contrast micrograph of BMMSC and BMMSC-sf. (g) Adipocyte differentiation of BMMSC and BMMSC-sf. (h) Oligodendroglial (GalC-immunoreactive) differentiation of BMMSC for 3 or 7 days. (i) Cytometric analysis of cell surface markers in BMMSC-sf of the first four passages (P). *P < 0.05 compared with P0.

Cultured BMMSC and BMMSC-sf, expanded in serum-containing and serum-free conditions, respectively, were first compared and characterized by flow cytometry. Figures 1(a), 1(b), 1(c), 1(d), and 1(e) showed that both cells were >90% immunoreactive to CD29, CD44, CD90, and CD54, major surface markers specific for BMMSC. By contrast, both cultures presented lower immunoreactivity (IR) to CD34, a surface marker for early hematopoietic stem cells. Phase-contrast microscope of cultured BMMSC and BMMSC-sf was shown in Figure 1(f). BMMSC exhibited healthy and fibroblast-like morphology, consistent with previous findings of BMMSC characteristics. By contrast, sphere formation could be observed in BMMSC-sf. These neurosphere-like structures were expanded for an additional 2–10 weeks (2–5 passages). Immunoreactivity to CD44, CD54, and CD34 (low) in BMMSC-sf was kept constant (>98%) regardless of cell passages (Figure 1(i)). CD29- and CD90-IR were decreased with later passages.  Figure 1(g) showed that both cultures showed potentials to differentiate oil-drops-containing adipocytes. Red color denoted oil drops in the cells. No significant difference of adipocyte differentiation was detected in both BMMSC and BMMSC-sf. By contrast, the differentiation of GalC-positive cells could be detected only in cultured BMMSC at day 3 and day 7 but not in cultured BMMSC-sf (Figure 1(h)).

**Figure 2:**
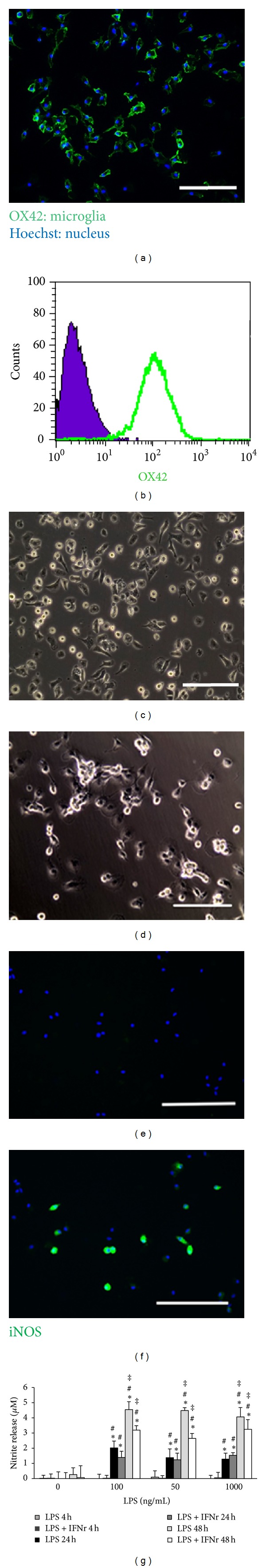
Identification of nonstimulated and LPS-stimulated microglia. (a) OX42-IR microglia counterstained with Hoechst 33258; (b) >98% cells were OX42-IR microglia analyzed by flow cytometry; (c) and (e) nonstimulated cultures and (d) and (f) LPS-stimulated cultures; (f) denotes iNOS-IR cells; (g) nitrite release to medium in LPS- or combined LPS and IFN*γ*-treated microglial cultures. LPS doses were ranging from 100 ng/mL to 1 *µ*g/mL. Aliquot of media was collected at 4, 24, or 48 hrs after treatment. Data were means ± SEM, *P < 0.05 compared with 0 ng/mL LPS (control) group. ^#^ *P* < 0.05 compared with 4 hrs treatment. ^‡^ *P* < 0.05 compared with 24 hrs treatment.

Microglial cultures were purified from mixed glial cultures which were prepared from neonatal rat brains. Characterization of resting and activated microglia was determined by both immunocytochemistry and flow cytometry. Almost all cells were OX42-IR microglia (Figure 2(a)). Flow cytometric results further demonstrated that these enriched cultures were >98% OX42-IR microglia (Figure 2(b)). Figures 2(c), 2(d), 2(e), and 2(f) showed the phase-contrast or iNOS-IR micrographs of nonstimulated (resting) or endotoxin LPS-stimulated microglia. Resting microglia did not express iNOS-IR (Figure 2). At 4 hours after LPS treatment, iNOS or nitrite was not detected in all treated groups. Not until 24 hours after LPS (or plus interferon *γ*) treatment, microglia were induced to express iNOS and release NO (in a form of nitrite) to cultured medium (Figures 2(f) and 2(g)). The nitrite levels in 100 ng/mL LPS and 100 ng/mL LPS-combined IFN-*γ* groups were 2.01 ± 0.44 *μ*M and 1.39 ± 0.4 *μ*M, respectively. Levels of NO release in 48 hours treatment groups were almost 2-fold higher than those of 24 hours treatment. However, similar levels of NO release were observed across LPS dosages (100 ng-1 *μ*g/mL). Furthermore, combined treatment of LPS with IFN-*γ* was not as powerful as LPS alone to activate microglia. Therefore, a dose of LPS 100 ng/mL and treatment for 24 hours were mainly employed for following microglia-BMMSC coculture studies.

**Figure 3:**
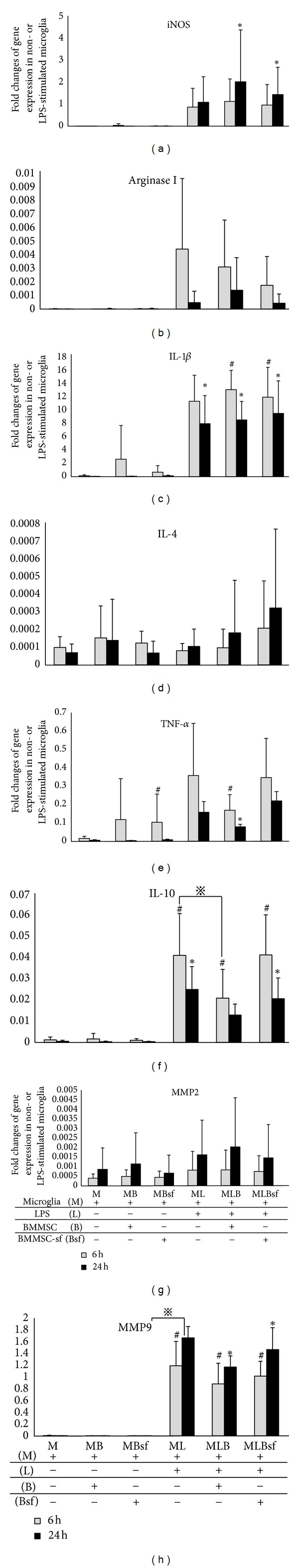
Quantitative changes of protein or cytokine expression in microglia cocultured with BMMSC or BMMSC-sf. Q-PCR results revealed that the expression of iNOS (a), IL1*β* (c), TNF*α* (e), IL-10 (f), and MMP-9 (h) was induced in LPS- (L-) treated microglia (M) or LPS-treated cocultures of microglia with BMMSC (b) or with BMMSC-sf (Bsf). By contrast, levels of arginase I, IL-4, and MMP-2 did not change among treatments. (a) iNOS was upregulated in LPS-stimulated MB or MBsf coculture for 24 hrs. The levels of IL-1*β* in (c) were upregulated by LPS stimulation at both 6 and 24 hrs. Higher expression levels of TNF-*α* in (e) and IL-10 in (f) were found at 6 hrs than at 24 hrs after LPS stimulation. BMMSC cocultured with microglia for 6 hr reduced LPS-stimulated IL-10 level. (h) MMP-9 levels were induced by LPS in all cultures. BMMSC cocultured with microglia for 24 hrs reduced LPS-stimulated upregulation. *y*-axis in (a)–(h) was the fold changes of gene expression in non- or LPS-stimulated microglia. Determinations are means ± SEM from RT-QPCR experiments. ^#^ *P* < 0.05 LPS LPS stimulation versus no LPS stimulation at 6 hrs treatment. *P < 0.05 LPS stimulation versus no LPS stimulation at 24 hrs.

The transwell culture system was utilized for indirect coculture of microglia and BMMSC. Microglia were seeded in transwell and pretreated with vehicle or 100 ng/mL LPS for 24 hours before being cocultured with BMMSC or BMMSC-sf. After further incubation for 24 hours in the presence or absence of LPS, microglial cells were harvested and processed for real-time PCR analysis for immune-related cytokines and proteins. As shown in Figure 3, the quantitative expression levels of iNOS, IL1*β*, TNF*α*, IL-10, and MMP-9 were induced in LPS-treated microglia or LPS-treated cocultures of microglia with BMMSC or with BMMSC-sf at 6 hours or 24 hours after treatment. By contrast, the expression levels of arginase I, IL-4, and MMP-2 did not change among treatments. iNOS was upregulated in LPS-stimulated coculture at 24 hours. The levels of IL-1*β* were upregulated in all cultures by LPS stimulation at both 6 and 24 hours. Interestingly, the expression levels of IL-1*β*, TNF-*α*, and IL-10 tended to be higher at 6 hrs than at 24 hours after LPS stimulation (Figures 3(c), 3(e), and 3(f)). BMMSC cocultured with microglia for 6 hours reduced LPS-stimulated IL-10 level. MMP-9 levels were induced by LPS in all cultures. On BMMSC coculture with microglia for 24 hours, MMP-9 level was significantly attenuated (Figure 3(h)).

**Figure 4:**
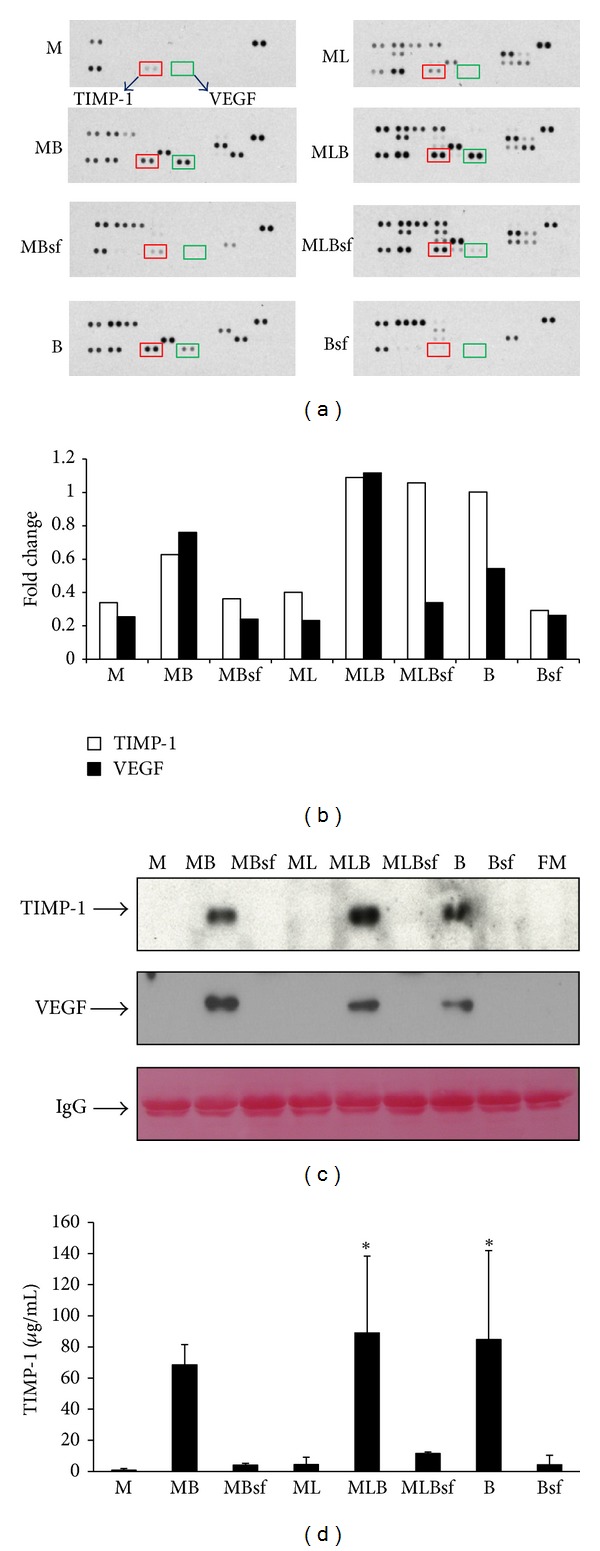
Identification of soluble factors released to conditioned media by microglia, BMMSC, and cocultures. (a) Expressions of 29 rat cytokines in the conditioned media. Spots of TIMP-1 (red square) and VEGF (green square) were found to change among treatments. (b) Histogram showing the fold changes of TIMP-1 and VEGF among treatments. (c) Western blot analysis of TIMP-1 and VEGF released in the 24 hrs conditioned medium of all cultures. The Ponceau-S staining (red) of IgG was used as an internal control. (d) Histogram showing the amounts, in *μ*g/mL, of the TIMP-1 as quantified by ELISA. Determinations were means ± SEM from coculture media subjected to ELISA experiments. *P < 0.05, compared to M group.

To evaluate the soluble factors released by microglia, BMMSC, or coculture, the media were harvested for cytokine array, western blot, and ELISA assays.  Figure 4(a) showed the array identification of 29 rat cytokines released to conditioned media by microglia, BMMSC, and cocultures. All spots were quantified by Image J densitometry software (Version 1.6, National Institutes of Health, Bethesda, MD). Interestingly, spots of TIMP-1 (tissue inhibitor of metalloproteinase-1; red square) and VEGF (vascular endothelial growth factor; green square) were found to change among treatments. Histogram in Figure 4(b) showed the fold changes of TIMP-1 and VEGF among treatments. Levels of TIMP-1 and VEGF release in the 24 hr conditioned media were further identified by western blot analysis. The Ponceau-S staining (red) of IgG was used as an internal control. Positive bands, corresponding to TIMP-1 and VEGF, were observed in the conditioned media of BMMSC and in microglia-BMMSC cocultures, but not in the media of BMMSC-sf or its coculture. Because TIMP-1 levels in the conditioned medium were not consistent between array and western blot analysis, we employed ELISA assay for it. Histogram in Figure 4(d) shows the amounts, in *μ*g/mL, of the TIMP-1 release, which was consistent with the results of western blot analysis. Therefore, TIMP-1 expression in the MLBsf group was low, not detected, or below detecting level.

## 4. Discussion

The central aim of the present study is to examine the effectiveness of bone marrow mesenchymal stem cells on microglial activation in a culture platform. BMMSC expanded in serum-containing and in serum-free medium were characterized and compared. BMMSC-sf were cultured in neurosphere-like structures according to methods similar to those propagation of neural stem cells [15, 16]. The phenotypes of these cells were similar to mesenchymal stem cells. Because BMMSC-sf exhibited sphere-like morphology, two adherent cell surface markers, CD29 and CD90, were reduced in BMMSC-sf when compared with BMMSC. Both cells maintained the capacity of mesodermal characteristics and multilineage differentiation. However, BMMSC differentiated faster into neural-like cells, such as GalC-positive cells.

The modulation of BMMSC on resting or activated microglia was revealed using a transwell coculture system that permits cell-contact-independent communication through diffusible soluble factors. Cellular gene expression of microglia and the released factors of cocultures were analyzed. BMMSC or BMMSC-sf cocultured with microglia did not alter the mRNA expression of iNOS, Arginase-1, IL-1*β*, IL-4, TNF-*α*, and MMP-2. However, the mRNA expression levels of IL-10 and MMP-9 in microglia were downregulated after being cocultured with BMMSC. The reduction of MMP-9 mRNA after cocultures might be due to TIMP-1 secretion from BMMSC.

TIMP-1 was present in the conditioned medium of BMMSC or BMMSC-microglia cocultures (Figure 4), analyzed by cytokine array and western blot. ELISA assay of TIMP-1 in the media further showed significant higher TIMP concentration in MB, MLB, and B groups than others.

Microglia were activated by LPS, a cell wall component of gram-negative bacteria and a powerful immune challenge associated with an increase of numerous cytokines. The LPS-induced generation of free radicals in microglia was an upstream event serving to regulate the production of other proinflammatory factors [17, 18]. The anti-inflammatory role of grafted BMMSC as a protective mechanism has been shown in* in vitro* and *in vivo* studies [19, 20]. Although the mechanism of the inhibitory effect of BMMSC on microglial activation was not clear, evidence suggests that microglial activation can be modulated by various cytokines and neurotrophic factors [7, 21]. In the present study, BMMSC decreased mRNA expressions of IL-10 and MMP-9 from microglia stimulated by LPS in a contact-independent manner. Furthermore, significant levels of TIMP-1 and VEGF were released from BMMSC, regardless of being in coculture or not. Therefore, it was speculated that soluble factors released from BMMSC might regulate the microglia response to LPS in our experiment. Reducing microglial activation by BMMSC would lend to neuronal protection against LPS stimulating cascade.

MMP-9 belonged to the family of extracellular calcium- and zinc-dependent proteinase that degraded the extracellular matrix and other extracellular proteins [22]. MMP-9 expression had been shown in the injured spinal cord [23], and BMMSC could reduce the MMP-9 expression in injured tissues [24]. Ample evidence shows that TIMPs regulate the activities of MMPs through protein-protein interaction. Both TIMPs and MMPs had the dynamic balance in activity [25]. Among at least four TIMP subgroups, TIMP-1 exhibits higher affinity for MMP-9 and thus effectively inhibits MMP activity. Consistent with this, the present results show that BMMSC released TIMP-1, thus reducing MMP-9 expression in microglia of coculture.

BMMSC have the abilities of self-renewing and differentiation into different types of cells. These properties make BMMSC suitable for tissue regeneration and cell therapy. In this study, we employed serum-containing and serum-free cultivation to expand adult bone marrow stem cells* in vitro*. Both cells expressed major surface markers specific for mesenchymal stem cells. In the beginning, we used fetal bovine serum for initial cell seeding and then switched to serum-free condition for BMMSC-sf expansion. This method was convenient to generate BMMSC-sf for further study. To avoid contamination of animal serum for BMMSCsf cultivation, patients' own serum might be applicable. Thus, BMMSC-sf might be promising materials for cell therapy in clinic.

In conclusion, we provided a platform for an *in vitro* assay to characterize the effects of BMMSC on LPS-stimulated microglia. Two different cultivated BMMSC were employed throughout, that is, BMMSC and BMMSC-sf. The BMMSC-sf had sphere morphology, having reduced expression of two adherent cell markers, CD29 and CD90. BMMSC exhibited typical fibroblast-like structure. BMMSC had faster differentiation potential into neural-like cells. BMMSC released significant levels of TIMP-1 and VEGF. Furthermore, it reduced mRNA expression of IL-10 and MMP-9 in microglia. This downregulated MMP-9 mRNA might be caused by TIMP-1 secretion from BMMSC in cocultures. Our cell culture system provided a powerful tool for investigating the molecular and cellular changes in microglia-BMMSC cocultures.
